# The Association of Human Herpesviruses with Malignant Brain Tumor Pathology and Therapy: Two Sides of a Coin

**DOI:** 10.3390/ijms22052250

**Published:** 2021-02-24

**Authors:** Evita Athanasiou, Antonios N. Gargalionis, Fotini Boufidou, Athanassios Tsakris

**Affiliations:** 1 Department of Biopathology, Eginition Hospital, National and Kapodistrian University of Athens, 115 28 Athens, Greece; evitathan@yahoo.gr (E.A.); fboufidou@med.uoa.gr (F.B.); 2 Department of Microbiology, Medical School, National and Kapodistrian University of Athens, 115 27 Athens, Greece; atsakris@med.uoa.gr

**Keywords:** glioma, brain cancer, CMV, Epstein–Barr, herpesvirus, immunotherapy

## Abstract

The role of certain viruses in malignant brain tumor development remains controversial. Experimental data demonstrate that human herpesviruses (HHVs), particularly cytomegalovirus (CMV), Epstein–Barr virus (EBV) and human herpes virus 6 (HHV-6), are implicated in brain tumor pathology, although their direct role has not yet been proven. CMV is present in most gliomas and medulloblastomas and is known to facilitate oncomodulation and/or immunomodulation, thus promoting cancer cell proliferation, invasion, apoptosis, angiogenesis, and immunosuppression. EBV and HHV-6 have also been detected in brain tumors and high-grade gliomas, showing high rates of expression and an inflammatory potential. On the other hand, due to the neurotropic nature of HHVs, novel studies have highlighted the engagement of such viruses in the development of new immunotherapeutic approaches in the context of oncolytic viral treatment and vaccine-based strategies against brain tumors. This review provides a comprehensive evaluation of recent scientific data concerning the emerging dual role of HHVs in malignant brain pathology, either as potential causative agents or as immunotherapeutic tools in the fight against these devastating diseases.

## 1. Introduction

Cancer of the central nervous system (CNS) refers to a heterogeneous group of non-communicable diseases that affect both adults and children. They are diagnosed in all anatomical regions of the CNS, with the vast majority (>90%) arising within the brain parenchyma and the rest appearing in the meninges, spinal cord, and cranial nerves [1,2,3]. They are associated with considerable morbidity and mortality worldwide. In 2016, there were 330,000 incident cases of CNS cancer and 227,000 deaths globally. Age-standardized incidence rates of CNS cancer have increased globally by 17.3% between 1990 and 2016 [1]. Gliomas account for almost 30% of all primary brain tumors and 80% of the malignant ones. Glioblastoma represents a type of high-grade glioma and is one of the most lethal cancers in adults [4]. Furthermore, medulloblastoma, an embryonal tumor of the cerebellum, is among the most common malignant childhood brain tumors with an annual incidence of almost five cases per one million individuals, and it has a poor prognosis [5].

To date, factors that may increase the risk of developing a brain tumor remain under investigation. Epidemiological studies have explored numerous such factors, including ionizing and non-ionizing radiation, toxic chemicals (*N*-nitroso compounds and pesticides), air pollution, smoking, diet, allergies, medications, endogenous and exogenous hormone exposure, genetic predisposition, and several demographic (age, sex, race, etc.) and anthropometric characteristics. Thus far, the only validated associations for brain tumors are ionizing radiation—even at low doses and especially during childhood—and a history of allergies [6,7].

A viral etiology of specific brain tumors has gained interest, although the role of viruses in oncogenesis remains controversial. White and colleagues reviewed the most common viruses that are likely to have a causative role in human cancer by inducing immunosuppression, modifying host cells through triggering oncoproteins, or altering the expression of host cell proteins at viral integration sites [8]. Among them, two herpesviruses, Epstein–Barr virus (EBV) and Kaposi’s sarcoma-associated herpesvirus (KSHV), are well-recognized for their oncogenic properties (EBV in Burkitt’s and Hodgkin’s lymphomas, as well as nasopharyngeal carcinoma, and KSHV in Kaposi’s sarcoma and primary effusion lymphoma) [8,9]. In brain cancer research, the experimental paradigms of brain tumor pathogenesis, which include the use of glial stem/progenitors, appear to be the most interesting in revealing the oncogenic potential of latent viruses, especially herpesviruses, which remain “the usual suspects” in the spotlight of current investigations [9]. Along this line, the progress in molecular methods and immunohistochemistry have made it possible to detect both viral genes and viral proteins in tumor tissue.

In this review article, we aim to summarize recent literature findings that indicate a potential causative link between herpesviruses and gliomas (the vast majority of malignant brain tumors), and to highlight the role of herpesviruses in brain tumor therapeutic prospects.

## 2. Herpesviruses as Potent Causative Agents in Brain Tumors

Herpesviruses are DNA viruses with a unique four-layered structure: a core containing the linear double-stranded DNA (with a length of 124–295 Kb) enclosed by an icosahedral capsid that is surrounded by an amorphous protein coat (the tegument), and a further glycoprotein-bearing lipid bilayer envelope [10]. Human herpesviruses (HHVs) are distributed worldwide, and more than 90% of adults are infected by one or multiple HHVs [11]. HHVs are categorized into three subfamilies: alpha (α), beta (β) and gamma (γ). Herpes simplex virus (HSV) 1 and 2, and varicella zoster virus (VZV), are α-herpesviruses, cytomegalovirus (CMV) and HHV-6 and -7 are β-herpesviruses, whereas EBV and KSHV are γ-herpesviruses. HHVs can cause a lytic infection or establish a latent infection within specific tissues characteristic for each virus. Among them, CMV, EBV and HHV-6 have been suspected to be strongly linked with tumorigenesis in the brain, although their direct role has not yet been proven.

### 2.1. CMV and Brain Tumors

Multiple reports illustrate the presence of CMV in different malignancies, including colon and gastrointestinal adenocarcinomas [12,13], breast cancer [14,15], and mucoepidermoid carcinoma of the salivary glands [16]. In terms of brain tumors, the presence and detection of CMV in gliomas and medulloblastomas is hotly debated. In 2002, Cobbs and colleagues were the first to show that CMV nucleic acids and proteins are present in a high percentage of low- and high-grade malignant gliomas, and that early and delayed CMV gene products are expressed in these tumors [17]. Almost a decade later, oncologists and virologists reached a consensus that CMV sequences and viral gene expression occur in most, if not all, malignant gliomas based on published data and ongoing research [18]. Furthermore, numerous studies reported evidence for the existence of CMV in medulloblastomas [19,20,21,22]. A recent meta-analysis of eleven studies revealed that when the major immediate early antigen was used as the detection index, the degree of infection was associated with the prognosis of glioma patients [23]. On the contrary, several studies have been published refuting the above findings [24,25,26]. Recently, researchers aimed to detect CMV in 36 infantile brain tumors other than glioblastoma multiforme, and viral DNA was not detected in any of these tumors [27]. Furthermore, comprehensive assessment of the brain virome in 111 GBM tumors revealed the absence of CMV [28]. It was stated that the reason for the conflicting findings lies in the lack of sensitivity in the methods used by the different research groups. In a recent study, Bartek and colleagues compared three detection methods used in the literature and clarified that the failure of some laboratories to detect CMV in medulloblastoma specimens most likely reflects technical problems, such as the use of inappropriate pH buffers and lack of sensitivity in immunohistochemistry methods for CMV protein detection [19].

Despite the contradictory reports showing the presence or absence of CMV in brain tumor tissues, there is an accumulation of evidence suggesting that CMV may play an oncomodulatory role in brain tumor progression. The term oncomodulation refers to the capability of non-coding RNAs and viral proteins to promote oncogenic processes through perturbations in different intracellular signaling pathways without provoking direct oncotransformation [9]. The CMV genome encodes about 250 proteins and 12 miRNAs, some of which possess oncomodulatory potential, affecting cell proliferation, cancer cell invasion, apoptosis, or autophagy, and promoting angiogenesis or immunosuppression [29]. Among them, CMV encoded chemokine receptor US28, protein pp71, CMV immediate early proteins and CMV70-3P miRNA have been shown to play pivotal roles in malignant brain tumor progression [17,30,31,32,33,34,35,36] (Table 1). It is also noteworthy that murine CMV potentiated glioblastoma growth by increasing pericyte recruitment and angiogenesis due to alterations in the secretome of CMV-infected cells in an in vivo model [31].

In addition to the oncomodulatory role of CMV, its viral products are also thought to exhibit immunomodulatory properties. It has been demonstrated that the release of CMV interleukin-10 (CMV IL-10), a homolog of human IL-10 that is associated with higher-grade gliomas and may exacerbate a tumor’s invasive potential, is induced upon the in vitro infection of glioma cancer stem cells by human CMV [37,38]. CMV IL-10 can bind to the human IL-10 receptor and activate the STAT3 transcription factor, which plays a key role in immune suppression and tumorigenesis, especially in gliomas [39,40].

### 2.2. EBV and Brain Tumors

Although the vast majority of research studies concerning the involvement of HHVs in brain tumorigenesis have focused on CMV, scientific attention has recently shifted to EBV and its role in glioma pathogenesis [41,42]. EBV infections are widely spread in the human population, occurring mostly in childhood or early adulthood with a subsequent lifelong persistence. In 1964, EBV was discovered in Burkitt’s lymphoma cells by Sir Anthony Epstein and colleagues, thus making it the first recognized human cancer virus [43]. The virus is primarily linked to epithelial cell cancers and lymphomas. Epithelial cancers, such as nasopharyngeal carcinoma and the nearly 10% of gastric carcinomas that are associated with EBV, outnumber in incidence the EBV-associated lymphomas, which include Burkitt’s lymphoma, Hodgkin’s lymphoma, diffuse large B cell lymphoma, natural killer (NK)/T cell lymphoma, and primary effusion lymphoma [44].

Regarding the CNS, EBV is primarily correlated with acute cerebellar ataxia and acute disseminated encephalomyelitis [45], while accumulating data support a robust association between EBV infection and multiple sclerosis pathology [46]. EBV has been implicated in the pathogenesis of primary CNS lymphomas in both immunodeficient and immunocompetent patients, with certain viral products (including the latent membrane protein 1 (LMP-1), Epstein–Barr virus nuclear antigen 2 (EBNA2), and EBV-encoded small RNAs (EBERs)) being blamed for their oncogenic properties [47,48]. Herman and colleagues identified 11 viral miRNAs that were differentially expressed in the plasma of patients with glioblastoma compared to those of healthy volunteers that induced signaling mostly related to impaired immune responses in tumor progression [49]. Six of these belonged to EBV miRNAs. Despite these findings, the association of EBV infection with other brain tumors, and mainly gliomas, is still debated [41].

In 2008, a study investigated the presence of the eight known HHVs in paraffin-embedded brain tissue biopsies from patients suffering from pilocytic astrocytoma using, for the first time, a specific real-time quantitative polymerase chain reaction (PCR). It revealed that EBV was the most frequent herpesvirus, but at levels obviously too low to be responsible for tumor initiation [50]. More recently, Cimino and colleagues detected EBV DNA in high grade gliomas (mostly glioblastomas) by applying a viral discovery pipeline to the analysis of unmapped next-generation sequencing (NGS) data, but the in situ hybridization results indicated the absence of a transcriptionally active virus, implying that EBV DNA is most likely found in gliomas due to the high levels of latent infection within the population [51]. However, by investigating the presence of HHV genome sequences in different grades of astrocytomas, another research group observed that EBV DNA was present at a significantly higher frequency in samples derived from patients with glioblastoma (astrocytoma grade IV) compared to controls obtained from autopsy tissue specimens which were not associated with neurological disease as the cause of death [52]. Fonseca and colleagues studied fresh frozen glioma tissues of different histological subtypes using conventional PCR, and they reported a 14.7% prevalence of EBV DNA in WHO (World Health Organization) grade III and IV gliomas [53]. Similarly, Stojnik and colleagues detected EBV DNA in three out of 45 tissues from glioblastomas, but none of the patients were found to be seropositive for EBV antibodies, possibly due to the very low amounts of the virus [54]. In line with the above findings, Zavala-Vega and colleagues detected LMP-1 by immunohistochemistry and EBER (Epstein-Barr virus encoded RNA) expression by in situ hybridization in patients with glioblastoma multiforme from Mexico [55]. On the other hand, research findings based on NGS casted doubt on the association of EBV with high grade gliomas [56], while several other studies depicted the total absence of EBV and other neurotropic viruses in gliomas [57,58,59].

### 2.3. HHV-6 and Brain Tumors

In 1986, HHV-6 was isolated for the first time from peripheral blood leukocytes of patients with lymphoproliferative disorders or AIDS (Acquired immunodeficiency syndrome), and was characterized as a novel human B-lymphotropic virus as it was morphologically similar to viruses of the herpesvirus family [60]. HHV-6 is the collective name for the double-stranded DNA viruses HHV-6A and HHV-6B that were formally classified as two separate species based on documented epidemiological, biological, and immunological distinctions [61]. HHV-6A infections are less characterized, presumably because this variant is acquired later in life when most people have already been infected with HHV-6B, an omnipresent virus that infects almost 90% of the world’s population in the first two years of life, known as the causative agent of exanthema subitum [62].

Like other HHVs, HHV-6 displays broad cellular tropism and has been detected in hematological, neurological, gastrointestinal, gynecological, and head and neck cancers [63]. In 2001, a study based on nested PCR was conducted to investigate the presence and variant distribution of HHV-6 in 118 biopsies from patients with a nervous tissue tumor and in 31 autopsy samples from the brains of healthy donors [64]. HHV-6 DNA sequences were identified in normal and neoplastic nervous tissue at a frequency of 32% and 37%, respectively (14 out of 31 patients with glioblastoma were HHV-6 positive), while the serological data indicated a similar frequency distribution of anti-HHV-6 antibodies in both patients and healthy donors. In another study, among 60 patients with invasive and non-invasive pituitary adenomas, HHV-6B DNA was detected in biopsy samples from 53.55% of invasive cases and 30% of non-invasive cases [65]. Crawford and colleagues screened 122 gliomas, 22 non-glial tumors, and 32 non-tumor autopsy controls derived from pediatric subjects for the presence of HHV-6 by in situ hybridization, nested PCR, and immunohistochemistry, and revealed higher rates of expression of the viral proteins U57 (major capsid protein) and U31 (large tegument protein) in tumors compared to non-tumor controls [66]. The same research group also examined the presence of HHV-6 in a large cohort of adult primary and recurrent CNS tumors and noticed that 47% of tumors were positive for U57, while 24% and 35% of tumors were positive for the HHV-6A/B early (p41) and late (gp116/64/54) antigens, respectively, suggesting active infection [67]. Moreover, HHV-6A/B early and late antigens were detected in glial tumors at a three-times higher frequency than in non-glial tumors, revealing a more frequent active infection among those with glial tumors. Using nested PCR, Chi and colleagues identified HHV-6 DNA in 17 out of 40 glioma tissue samples, compared to only one out of 13 control samples [68]. They also managed to isolate a HHV-6 strain from cyst fluid specimens obtained from a patient with glioma and demonstrated that HHV-6 infection can provoke proinflammatory cytokines in cyst fluids of patients with glioma and in astrocyte cultures that might provide a chronic inflammatory environment to facilitate the development of glioma. In line with these findings, a recent study depicted the expression of HHV-6-encoded DR7 in 13 out of 27 glioma tissues, and only in 5 out of 30 normal brain tissues [69]. This protein has been reported to possess malignant transforming activity and be involved in Hodgkin’s lymphoma carcinogenesis [70,71]. Another HHV-6 protein, U24, expressed in the early stages of infection [72], is newly recognized as a cognate ligand for the human neural precursor cell expressed developmentally down-regulated protein 4-like (HNedd4L-WW3) domain, whose dysregulation has been observed in gliomas [73,74].

The in vitro and in vivo scientific data supports that both HHV-6A and HHV-6B are present in gliomas. The higher prevalence of HHV-6 DNA and proteins in glial tumors compared to controls and the altered cytokine profile in HHV-6-positive specimens indicate a possible role of the virus in tumorigenesis [63], however confirmation is still lacking [75].

## 3. Therapeutic Approaches against Malignant Brain Tumors That Take Advantage of HHVs Neurotropism

The current standard of care for patients with malignant brain tumors consists of complete or maximal surgical resection followed by targeted radiation therapy and chemotherapy such as temozolomide (TMZ). Although many patients benefit from multimodal therapy combining the above treatment options, they often sustain long-term complications such as neurosensory and neurocognitive impairments [76], myelosuppression [77], and opportunistic infections [78]. Further, malignant gliomas are notably resistant to the current standard of care and continue to be a leading cause of cancer-related mortality in both children and adults. Thus, there is a critical need for more effective treatment strategies, and immunotherapy, among others, has attracted research interest as a challenging but highly promising option. Despite the fact that the theory of HHVs involvement in the development of malignant brain tumors remains in question, the neurotropic nature of HHVs could be the basis for new immunotherapeutic approaches against these devastating diseases.

### 3.1. Oncolytic Viral Therapy for Brain Tumors

As normal cells evolve progressively to a neoplastic state, they acquire a succession of hallmark capabilities which include sustaining proliferative signaling, evading growth suppressors, resisting cell death, enabling replicative immortality, reprogramming of energy metabolism, and evading immune destruction [79]. Considering the last hallmark, tumor cells are able to avoid immune-mediated recognition and destruction by disabling components of the immune system that have been dispatched to eliminate them. While the concept of CNS immune privilege has eroded over time, and the notion that the blood brain barrier acts as a “hermetic seal” to immune cell entry has been disproved, brain tumors still possess the ability to prevent immunity and facilitate their own modes of immune evasion [80].

Since antiviral defense responses found in normal cells (such as interferons) are usually inactivated in cancer cells as part of the malignant phenotype, theoretically, malignant cells are more susceptible to infection by at least some viruses. This natural propensity has been explored as an emerging anti-cancer immunotherapy by the exploitation of oncolytic viruses (OVs) [81,82,83]. OVs are naturally cancer-selective or can be genetically modified to reduce pathogenicity, increase lytic potential, and induce innate and adaptive anti-tumor immunity in the host [81,84]. The virus activates the local innate immune response, whereas the tumor-associated antigens released by lysed tumor cells prime the systemic adaptive anti-tumor immunity capable of targeting uninfected cancer cells at distant sites [84].

Herpes simplex virus type 1 (HSV-1) is well known as the causative agent of encephalitis, corneal blindness, and several other disorders of the peripheral nervous system and presents as a structurally complex enveloped double stranded DNA virus that replicates in peripheral tissues and then invades the nervous system to establish latency [85]. HSV-1 was the first genetically engineered virus to combat cancer. In 1991, a preclinical study reported that a genetically modified HSV-1 with a depleted thymidine kinase gene could hinder the growth of glioma in mice and prolong their overall survival with excellent safety [86]. Talimogene laherparepvec (T-VEC), an oncolytic HSV-1, is currently the only oncolytic virus approved by the Food and Drug Administration (FDA) for advanced melanoma treatment [87]. In light of this approval, HSV-1 has been intensively studied and accounts for nearly a quarter of all ongoing clinical trials using oncolytic viruses targeting various other cancer types except melanoma, including gastrointestinal cancer, breast cancer, and brain tumors, both as a single agent and in combination with other anti-cancer therapies [81]. Multiple clinical trials using oncolytic HSVs to treat malignant tumors of the CNS in children and adults are underway or have been completed [88] (Table 2). Cyclic GMP-AMP synthase (cGAS) has been identified as a major innate immune sensor for cytosolic DNA derived from a wide range of pathogens—including HSV—as well as tumor cells, and activates the stimulator of interferon (IFN) genes (STING) to induce the expression of type I IFNs and other immune modulatory molecules [89]. A recent study by Froechlich and colleagues demonstrated that a functional cGAS/STING pathway was essential to trigger anti-tumor adaptive immune responses in immunocompetent preclinical models, and thus it was considered a prerequisite for sustaining the immunotherapeutic efficacy of oncolytic HSVs [90]. This finding provided strong evidence that oncolytic virotherapy induces a tumor immune remodeling that overcomes the therapeutic effect of oncolytic HSVs, highlighting the antitumor benefit of antiviral immunity [90,91], and consequently oncolytic virotherapy could be a promising tool in the fight against non-immunogenic malignant brain tumors.

Recently, a beneficial anti-tumoral effect of CMV infection has been described in human and animal models through modulation of the tumor microenvironment with different mechanisms, including the induction of cell death, stimulation of cytokines and chemokines, interference with tumor cell extravasation or tumor vascularization, or bystander stimulation of an anti-tumoral immune response [92]. Thus, CMV offers several advantages and could be exploited as another promising oncolytic virus alone or in combination with other therapeutic approaches, such as immune checkpoint inhibitors or epigenetic modifiers. Further, in 2007, Shah and colleagues reported an enhanced anti-glioma activity of the chimeric human CMV/HSV-1 oncolytic viruses that have been constructed with the disruption of the HSV γ_1_34.5 neurovirulence gene to eliminate its ability to cause encephalitis, along with human CMV protein kinase R evasion genes IRS1 or TRS1, to enhance anti-tumoral activity [93]. The transfer of the human CMV IRS1 or TRS1 gene into the HSV γ_1_34.5 gene significantly improved replication in malignant gliomas without rebuilding wild type neurovirulence, leading to enhanced tumor reduction and prolonged survival. In an in vivo model of glioblastoma multiforme, human CMV/HSV-1 oncolytic virotherapy elicited an immune response characterized by the significant induction of CD8^+^ T cells, but not CD4^+^ T cells [94]. Moreover, this model revealed that a durable circulating immune memory against tumors was established in long-term survivors and that repeated administration of the CMV/HSV-1 oncolytic virus could extend its anti-tumor effects. Last but not least, chimeric human CMV/HSV-1 viruses induced infectivity and cytotoxicity in adult and pediatric patient-derived glioblastoma xenografts under hypoxia, a well-known pathophysiological marker of high-grade gliomas associated with tumor development, invasiveness, and resistance to radiation and chemotherapy [95].

The link between anti-viral innate immune responses induced by the OVs and anti-tumor adaptive immune responses raised the idea of combining OVs and immune checkpoint inhibitors to combat cancer, since OVs create a more immunogenic local tumor microenvironment that enhances the potential of checkpoint therapy [96,97,98,99]. A phase 1b trial in patients with advanced melanoma revealed that the combination of T-VEC and the anti-PD-1 antibody prembolizumab may be able to overcome some limitations of either single-agent therapy and provide stronger anti-tumor responses [100]. Considering malignant brain tumors, the addition of PD-1 blockade to the intravenous infusion of oncolytic human *Orthoreovirus* improved systemic therapy in a preclinical glioma model [101]. Further, the triple combination of an oncolytic HSV expressing IL-12 and two immune checkpoint inhibitors (anti-PD-1 and anti-CTLA-4 antibodies) demonstrated a great capacity to safely overcome the immune suppressive microenvironment and eliminate tumors in an immunocompetent model of glioblastoma [102]. Taking the above into account, the development of combined systemic approaches based on oncolytic virotherapy and immune checkpoint blockade represents an emerging research field in the context of brain tumor treatment.

### 3.2. Vaccine-Based Strategies against Brain Tumors

The study of tumor-specific antigens (TSAs) as potent targets for anti-tumor therapy has accelerated within the past decade, and part of it has been their exploitation for the development of TSA-based therapeutic vaccines [103,104]. Among the TSAs, viral-derived cancer antigens have gained scientific attention and the presence of CMV-specific antigens in malignant brain tumors has formed the basis for their use in anti-tumor vaccine strategies.

Different research groups have shown that CMV phosphoprotein 65 (pp65) RNA is expressed in more than 90% of glioblastoma specimens, but not in the surrounding normal brain tissue [17,18,105], providing a promising tumor-specific target. In 2014, Nair and colleagues demonstrated that CMV-specific T cells recognized and effectively killed autologous glioblastoma cells expressing viral pp65 at endogenous levels, supporting the rationale for the clinical evaluation of CMV-directed immunotherapy in patients with malignant gliomas [106] (Table 1). In more detail, CMV pp65 RNA-pulsed dendritic cells (DCs) were generated from the peripheral blood of patients with newly diagnosed glioblastoma, and stimulated CMV-specific CD4^+^ and CD8^+^ effector T cells were able to kill autologous tumor cells and autologous DCs pulsed with total tumor RNA with a CMV pp65-restricted killing mechanism. In a small randomized pilot trial, patients who received CMV pp65-specific DCs combined with tetanus–diphtheria toxoid preconditioning of the vaccine site presented with remarkably improved progression-free survival (PFS; range: 15.4–47.3 months) and OS (overall survival) (range: 20.6–47.3 months) compared to controls [105]. Two years later, in a phase I trial (NCT00639639), the same research group evaluated the safety and feasibility of vaccinating newly diagnosed glioblastoma patients with pp65-DCs admixed with GM-CSF (Granulocyte-macrophage colony-stimulating factor) following host conditioning with dose-intensified TMZ, as well as investigating patient cellular immune responses by determining PFS and OS and comparing them with those resulting from the current standard-of-care therapy [107]. Despite profound lymphopenia and increased T_reg_ (regulatory T cells) proportions following dose-intensified TMZ, patients receiving pp65-DCs showed an expansion of antigen-specific immunity and prolonged PFS (median: 25.3 months) and OS (median: 41.1 months).

Further, the fact that CMV infection contributes to the accumulation of functional antigen-specific CD8^+^ T cells with an effector memory phenotype served as the backbone for the development of a novel therapeutic vaccine platform based on human CMV vectors against different cancer types, including brain tumors [108,109]. In a recent preclinical study, a CMV-based therapeutic vaccine expressing the E6 peptide of human papillomavirus type 16 as a neo-epitope and lacking immunoevasins was generated in order to use the viral vector as an adjuvant for the presentation of endogenous tumor antigens and finally to repurpose bystander CMV-specific CD8^+^ T cells against the tumor [110]. Glioblastoma cells infected with these vectors were shown to be capable of efficiently stimulating E6-specific T cells.

### 3.3. Adoptive Cell Therapy

Within the context of cancer immunotherapy, adoptive cell therapy comprises the extraction of native T cells from the patient in order to be genetically modified so that upon their return into the individual, they can target and kill cancer cells specifically via the recognition of specific antigens expressed on the cancer cell surface. The two major strategies to generate tumor-specific T cells involve either altering the antigen-binding sites of the T-cell receptor (TCR) which require major histocompatibility complex (MHC)-dependent antigen presentation, or engineering a novel recombinant receptor, the chimeric antigen receptor (CAR), that activates T cells in an MHC-unrestricted manner, bypassing the downregulation of MHC complexes caused by glioma cells both on tumor and antigen-presenting cells [111].

In 2012, a study by Crough and colleagues revealed that the in vitro stimulation of CMV-specific T cells from glioblastoma patients with CMV peptide epitopes enabled them to produce high levels of multiple cytokines (macrophage inflammatory protein (MIP)-1β, tumor necrosis factor (TNF)α, and IFNγ) and enhance their cytolytic function (CD107a mobilization) [112]. Adoptive transfer of these in vitro expanded T cells combined with TMZ therapy into a single patient with recurrent glioblastoma was associated with long-term disease-free survival. Following these findings, a phase I clinical trial including 19 patients with recurrent glioblastoma provided strong evidence that autologous CMV-specific T cell therapy is safe with minimal side effects and may offer a prolonged clinical benefit [113]. A phase I/II clinical trial enrolling 65 patients with glioblastoma is still underway to investigate the effectiveness, side effects, and optimal dose of autologous CMV-specific cytotoxic T cells when provided in combination with TMZ (NCT02661282). Furthermore, a recent study based on a randomized pilot trial in patients with newly-diagnosed glioblastoma reported the safety and usefulness of using DCs pulsed with CMV pp65 RNA to enhance the multifunctionality of adoptively transferred CMV pp65-specific T cells [114].

Among adoptive cell therapeutic approaches in the fight against malignant brain tumors, the most prominent seems to be the CAR T cell technology, which was approved in 2017 by the FDA for the treatment of hematological malignancies with exciting results reported from several recent clinical trials [115]. Various cell surface proteins have been actively targeted, including epidermal growth factor receptor variant III (EGFRvIII), human epidermal growth factor receptor 2 (HER2), interleukin receptor 13Rα2 (IL-13Rα2), and erythropoietin-producing hepatocellular carcinoma A2 (EphA2) [116]. The expression of CARs in virus-specific T cells could enhance the persistence of adoptively transferred T cells, since not only do they provide antitumor activity through their CAR, but they may also receive appropriate co-stimulation following native TCR engagement by latent virus antigens presented by professional antigen-presenting cells [117]. Additionally, 17 patients with progressive HER2-positive glioblastoma were treated in a phase 1 trial with peripheral blood infusions of autologous T cells specific for CMV, EBV, or adenovirus and genetically modified to express HER2-CARs [118]. This trial demonstrated the feasibility and safety of peripherally infused virus-specific CAR T cells in glioblastoma, coincident with clinical benefit for patients (Table 2).

## 4. Concluding Remarks and Future Perspectives

**Figure 1:**
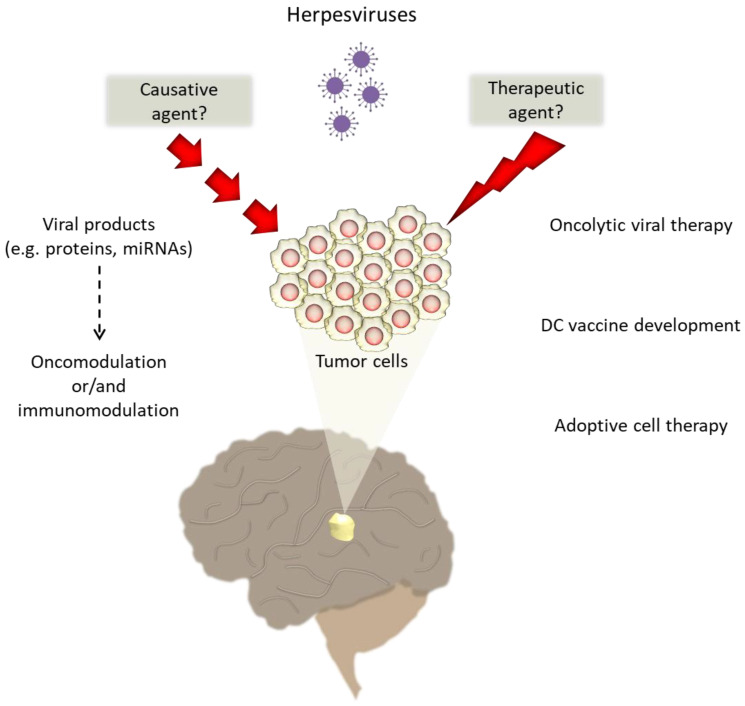
The interplay between herpesviruses and malignant brain tumors. The oncogenic, oncomodulating, or/and immunomodulating role of neurotropic herpesviruses in brain tumor development has been hotly debated and has not yet been proven. However, their neurotropic nature has formed the basis for the development of novel immunotherapeutic approaches.

Beyond ionizing radiation exposure and a history of allergies, no other well-established risk factors have been associated with brain tumor etiology. During many years of cancer research, accumulated evidence strongly supports the involvement of several viruses in human oncogenesis, affecting pathways relevant to the “hallmarks of cancer”. Infections are associated with the vast majority of CNS cancers and may modulate cancer pathogenesis, but it is unclear whether the infectious environment predisposes the patient to carcinogenesis by downregulating the host immune system or whether cancer cells debilitate the immune response so that the infectious agents can easily overcome the defense mechanisms and evade the host immune cells [131]. The well documented oncogenic potential of EBV and KSHV in lymphomas or carcinomas and the neurotropic properties of HHVs triggered numerous studies aiming to investigate HHV prevalence in malignant brain tumors, as well as their oncomodulating capacity. CMV, EBV, and HHV-6 have attracted most of the research interest, with a great number of conflicting reports claiming their presence or absence in brain tumor tissues (Table 3). Although both experimental and clinical data continue to be collected using various approaches by different research groups, other studies have revealed several viral products, such as viral encoded proteins and miRNAs, that seem to play an oncomodulatory or immunomodulatory role in brain tumor progression and may be useful as promising targets in immunotherapeutic approaches (Figure 1).

Relapse and resistance to standard-of-care treatment options represent one of the most prominent challenges faced by both researchers and clinicians in the field of malignant brain tumors. Immunotherapy has gained increasing interest, since it harnesses the patient’s innate and adaptive immune responses to attack and destroy tumor cells. However, its efficacy is significantly restrained in malignant brain tumors since these tumors tend to have few mutations that could be targeted immunotherapeutically. Furthermore, malignant brain tumors rarely have even one mutation that is expressed homogeneously, while the immunosuppression caused by standard therapies can thwart immunotherapeutic attempts [80]. Thus, there is an urgent need for the development of novel successful immunotherapies able to provide effective delivery and target specificity. HHVs are promising tools in this field of research, broadening the treatment options for patients diagnosed with these life-threatening diseases. Experimental studies and clinical trials have already documented the benefits of HHV use in immunotherapeutic strategies such as oncolytic virotherapy, peptide or DC vaccine development, and adoptive cell therapy (Figure 1). Regardless of a potent etiologic role in brain tumor pathology, extensive study of these neurotropic viruses and their oncomodulatory or immunomodulatory properties could provide new avenues for the effective treatment of malignant brain tumors.

## Figures and Tables

**Table 1:** The oncomodulatory role of cytomegalovirus (CMV) in malignant brain tumors.

CMV Product	Oncomodulatory Role	Supporting Evidence
US28	Activation of several proliferative, inflammatory and angiogenic signaling pathways through binding human chemokines [33,36]	Growth acceleration of glioblastoma cells in a murine orthotopic intracranial glioblastoma model [30]; VEGF suppression by RNA silencing of US28 in glioma cells [32]
pp71	Activation of endothelium tube formation [32]	Enhanced cancer cell proliferation in CD133^+^ glioma-initiated cells [32]
Immediate early proteins, CMV70-3P miRNA	Stemness of glioma-initiated stem cells	Increased expression of the stemness markers *sox2* and *nestin* in glioblastoma cells [34,35,41]

**Table 2:** Clinical trials in brain tumors that exploit immunotherapeutic strategies and the neurotropism of herpesviruses.

Therapy	Cancer Type	Phase	Status	Clinical Trial Identifier	Reference
**Oncolytic Viruses**					
G207 (genetically engineered HSV type I)	Glioma, astrocytoma, glioblastoma	I/II	Completed	NCT00028158	[119]
G207 (genetically engineered HSV type I)	Glioblastoma	I	Completed	NCT00157703	[120]
G207 (genetically engineered HSV type I)	Malignant cerebellar brain tumors	I	Recruiting	NCT03911388	[121]
G207 (genetically engineered HSV type I)	Malignant supratentorial brain tumors	I	Active	NCT02457845	[122]
M032 (second generation genetically engineered HSV type I)	Glioblastoma multiforme, anaplastic astrocytoma, gliosarcoma	I	Recruiting	NCT02062827	[123]
C134 (genetically engineered HSV type I)	Glioblastoma multiforme, anaplastic astrocytoma, gliosarcoma	I	Active	NCT03657576	[124]
G207 (genetically engineered HSV type I)	Malignant high-grade glioma	II	Not yet recruiting	NCT04482933	[125]
**Peptide and Dendritic Cells Vaccines**					
PEP-CMV (synthetic long peptide of 26 amino acid residues from human pp65)	Medulloblastoma, malignant glioma	I	Recruiting	NCT03299309	[126]
Nivolumab combined with CMV pp65 DC vaccination	Grade III or IV glioma or astrocytoma	I	Completed	NCT02529072	[127]
CMV pp65 DC vaccination combined with GM-CSF	Glioblastoma multiforme	I	Active	NCT00639639	[107]
CMV RNA-loaded DC vaccine	Glioblastoma	II	Recruiting	NCT03927222	[128]
CMV RNA-loaded DC vaccine +/– varlilumab	Glioblastoma	II	Recruiting	NCT03688178	[129]
**Adoptive C** **ell T** **herapy**					
Human epidermal growth factor receptor type2-Chimeric antigen receptor (HER2-CAR) virus specific T cells	HER2-positive glioblastoma	I	Completed	NCT01109095	[130]

**Table 3:** Findings regarding the controversial role of herpesviruses in malignant brain tumors.

HHVs (Human Herpesviruses)	Role
**CMV**	Detection of nucleic acids and proteins in a high percentage of malignant gliomas and medulloblastomas
	Absence in gliomas and tumor cells of the nervous system
	Oncomodulation by viral proteins and non-coding RNAs
	Immunomodulation by viral proteins and non-coding RNAs
**EBV (Epstein-Barr virus)**	Detection of EBV DNA in high-grade gliomas
	Presence in pilocytic astrocytomas
	EBV miRNAs in plasma of glioblastoma patients associated with impaired anti-tumor immune response
	Absence in gliomas
**HHV-6**	Detection of viral DNA and viral proteins in adult and pediatric gliomas compared to healthy controls

## Data Availability

Not applicable.
